# Utilisation of existing community rehabilitation services by critical care survivors

**DOI:** 10.1186/cc14637

**Published:** 2015-03-16

**Authors:** CR Soulsby, J McPeake, C Ashcroft, J Kinsella, M Shaw, T Quasim

**Affiliations:** 1 University of Glasgow, UK

## Introduction

Patients recovering from critical illness suffer many physical and psychological problems during their recovery, including muscle weakness, fatigue, signs and symptoms of PTSD, anxiety and depression [1]. At present, specialist intensive care follow-up and rehabilitation is inconsistent and in many geographical areas is nonexistent. As a result, many survivors of critical illness will require using existing community rehabilitation services [2]. The aim of this present service evaluation was to understand the utilisation of community rehabilitation services by critical care survivors.

## Methods

A database of acute referrals to community rehabilitation services was retrospectively analysed from 1 May 2014 to 31 October 2014. Age, referring specialty and reason for referral for rehabilitation were documented. This database was cross-checked with the critical care database in Glasgow Royal Infirmary to identify which individuals had been admitted to critical care during their admission.

## Results

Over this 6-month period 769 patients were referred from their parent specialty for community rehabilitation in North East Glasgow. Thirty-three of the 769 patients (4.3%) referred had a critical care stay during their admission. Of these, eight patients were referred for rehabilitation by orthopaedics, eight by medicine for the older patients, 11 from acute medicine and the remaining six from other specialties. Six of the 769 patients who had a critical care admission were of working age (<1%). Two individuals were admitted to critical care following trauma whilst four had complex social needs prior to their critical care admission. This included an individual with a high body mass index. None of the individuals of working age were referred as a consequence of their critical care stay.

## Conclusion

This service evaluation demonstrates that very few critical care survivors are referred to community rehabilitation services, particularly those of working age. More work is required to understand optimal rehabilitation pathways in this patient group.
